# Deiters Cells Act as Mechanical Equalizers for Outer Hair Cells

**DOI:** 10.1523/JNEUROSCI.2417-21.2022

**Published:** 2022-11-02

**Authors:** Wenxiao Zhou, Talat Jabeen, Sultan Sabha, Jonathan Becker, Jong-Hoon Nam

**Affiliations:** ^1^Departments of Mechanical Engineering; ^2^Biomedical Engineering, University of Rochester, Rochester, New York 14627; ^3^Neuroscience Program, University of Rochester Medical Center, Rochester, New York 14627

**Keywords:** basilar membrane, Deiters cell, organ of Corti, outer hair cell, reticular lamina, tectorial membrane

## Abstract

The outer hair cells in the mammalian cochlea are cellular actuators essential for sensitive hearing. The geometry and stiffness of the structural scaffold surrounding the outer hair cells will determine how the active cells shape mammalian hearing by modulating the organ of Corti (OoC) vibrations. Specifically, the tectorial membrane and the Deiters cell are mechanically in series with the hair bundle and soma, respectively, of the outer hair cell. Their mechanical properties and anatomic arrangement must determine the relative motion among different OoC structures. We measured the OoC mechanics in the cochleas acutely excised from young gerbils of both sexes at a resolution fine enough to distinguish the displacement of individual cells. A three-dimensional finite element model of fully deformable OoC was exploited to analyze the measured data in detail. As a means to verify the computer model, the basilar membrane deformations because of static and dynamic stimulations were measured and simulated. Two stiffness ratios have been identified that are critical to understand cochlear physics, which are the stiffness of the tectorial membrane with respect to the hair bundle and the stiffness of the Deiters cell with respect to the outer hair cell body. Our measurements suggest that the Deiters cells act like a mechanical equalizer so that the outer hair cells are constrained neither too rigidly nor too weakly.

**SIGNIFICANCE STATEMENT** Mammals can detect faint sounds thanks to the action of mammalian-specific receptor cells called the outer hair cells. It is getting clearer that understanding the interactions between the outer hair cells and their surrounding structures such as the tectorial membrane and the Deiters cell is critical to resolve standing debates. Depending on theories, the stiffness of those two structures ranges from negligible to rigid. Because of their perceived importance, their properties have been measured in previous studies. However, nearly all existing data were obtained *ex situ* (after they were detached from the outer hair cells), which obscures their interaction with the outer hair cells. We quantified the mechanical properties of the tectorial membrane and the Deiters cell *in situ*.

## Introduction

The organ of Corti (OoC), the sensory epithelium of the mammalian cochlea, vibrates in distinct patterns (modes) depending on the active mechanical feedback from the outer hair cells. In the passive form, the OoC turns transepithelial fluid pressures into vibrations of the hair cell stereocilia. For this purpose, the OoC must be stiff enough to deliver basilar membrane vibrations to the stereocilia. In the active form, the OoC takes and leverages outer hair cell motility to amplify the vibrations of the hair cell stereocilia. For this, the OoC must be compliant enough to be deformed by outer hair cells. Identifying mechanical attributes dictating the passive and active vibration modes of the OoC is pertinent to understanding the operating principles of cochlear amplification.

The OoC sits between two acellular matrices, the tectorial and the basilar membranes. The stiffness gradient of the basilar membrane is essential to explain cochlear traveling waves. Most measurements of basilar membrane stiffness were performed using calibrated probes, and then functionally relevant volume compliance was estimated (Naidu and Mountain, 1998; Emadi et al., 2004). The tectorial membrane is the structure against which the stereocilia of hair cells deflect. Depending on its attachment (radial) stiffness, either the stiffness or the inertia of the tectorial membrane contributes more to the reaction force at the stereocilia tips (Zwislocki and Kletsky, 1979; Meaud and Grosh, 2014). Note, that in most previous studies, tectorial membrane stiffness was measured after isolating the tectorial membrane from the OoC.

The outer hair cells are surrounded by mechanically significant structures. The stereocilia tips of outer hair cells are attached to the overlying tectorial membrane. The Deiters cell supports the outer hair cell base like a cup holder. The phalangeal process of the Deiters cell reaches the apex of other outer hair cells two to four cell distances apart, forming a truss-like structure together with the outer hair cell (Nam, 2014; Soons et al., 2015). The reticular lamina clamps outer hair cells at their stiffest part—the cuticular plate densely packed with actin filaments. Consequently, the reticular lamina separates outer hair cell mechanics into two parts. The stereocilia and the cell body of an outer hair cell deform differently reflecting their distinct functions. The stereocilia bend (deflect) about their thin rootlets to activate mechanotransduction, whereas the cell body actively contracts and relaxes to modulate the OoC vibrations. The tectorial membrane and the Deiters cell are mechanically in series with the mechanotransduction organelle (stereocilia bundle) and with the actuator for cochlear amplification (outer hair cell body), respectively. Because of such mechanical connectivity, the tectorial membrane, the reticular lamina, and the Deiters cell must dominate the stiffness felt by the outer hair cell.

Because mechanics determine basic cochlear functions such as cochlear tonotopy, tuning, and amplification, the mechanical properties of hair cells and their surrounding structures have been investigated. The stiffnesses of the hair cell stereocilia and body range from a few to tens of mN/m (Adachi and Iwasa, 1997; Beurg et al., 2008; Nam et al., 2015). The tectorial membrane could contribute to determining the best responding frequency (BF; Teudt and Richter, 2014) or to shaping traveling waves (Ghaffari et al., 2007). In most theoretical studies, the mechanical properties of Deiters cell were not incorporated explicitly, despite their presumed role in force transmission from the outer hair cells (Yoon et al., 2011; Nam, 2014; Sasmal and Grosh, 2019).

This study aimed to explain how the OoC achieves the two functional requirements stiff and compliant OoC for passive and active mechanics, respectively. To address this question, some rudimentary properties of OoC mechanics that have been tricky to acquire were quantified, such as the OoC complex volume compliance measured at the basilar membrane, the attachment stiffness of the tectorial membrane, and the stiffness of the Deiters cell.

From young gerbils, cochleas were acutely isolated and placed in a custom-designed microchamber. The OoC in the microchamber was subjected to either hydrostatic or hydrodynamic fluid pressures. Resulting deformations and vibrations of the OoC were measured using optical coherence tomography (OCT). The measured data were compared with simulations of a custom-written finite element (FE) model. The active and passive deforming patterns were reproduced by computer simulations incorporating identified mechanical properties.

## Materials and Methods

### Tissue preparation

**Figure 1.**
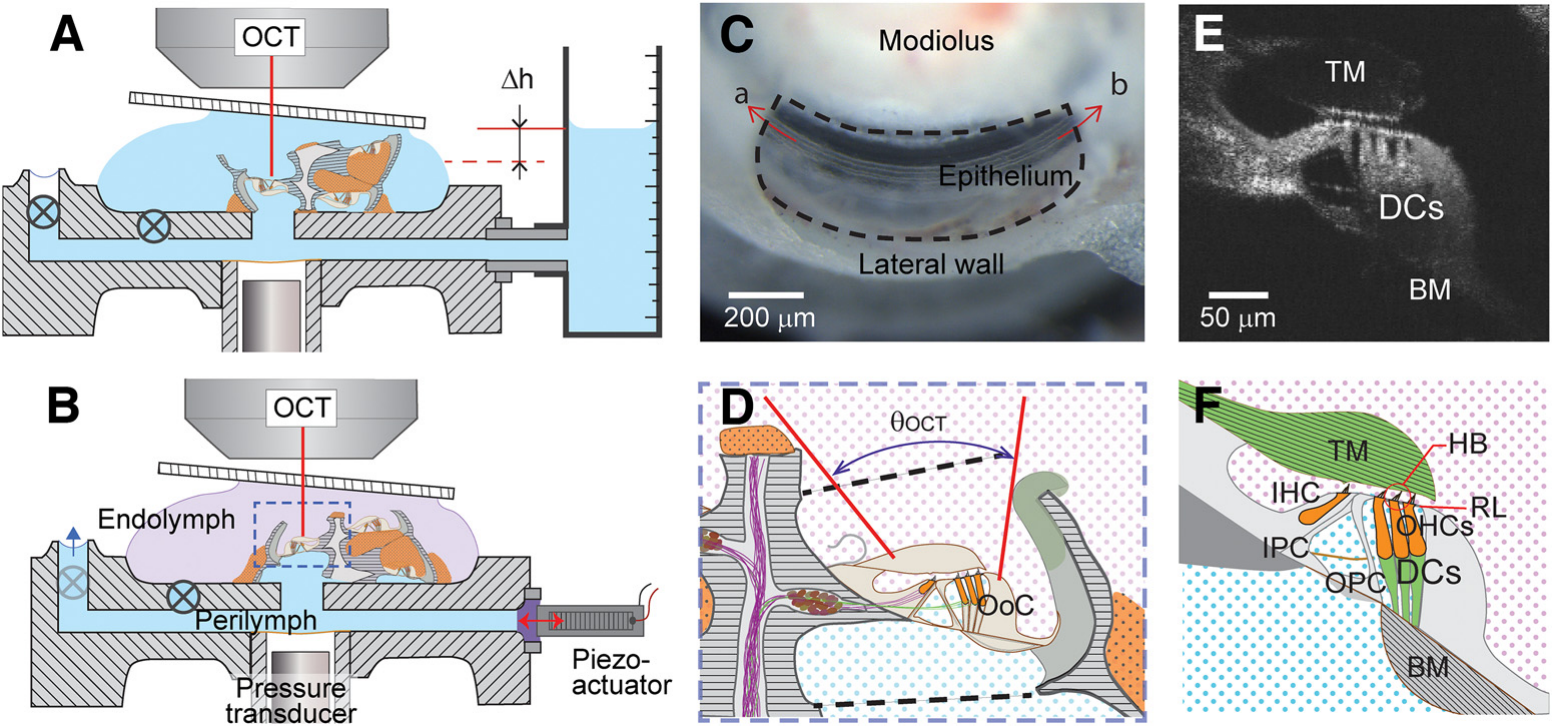
Experimental approach. ***A***, Preparation for hydrostatic measurements. Reduced cochlea placed in custom-designed microfluidic chamber. The basilar membrane faced upward. One fluid circulation channel was connected to a fluid vessel where the fluid level Δh was adjusted. Two valves (symbol ⊗) were closed after equalizing fluid levels between two fluid surfaces. Diagram is not to scale with exaggerated cochlear size. ***B***, Preparation for hydrodynamic measurements. Mechanical stimulations were delivered by piezoelectric actuator. A hydrophone was placed beneath the tissue. Left, Blue arrow at top left opening indicates a pressure release (faint ⊗ indicates open valve). ***C***, View of the sensory epithelium under dissection scope after the removal of apical and basal turns. The interscala bone was removed to expose the epithelium. The direction toward the apex or the base is indicated by a and b. ***D***, A closer view of the prepared tissue in the chamber. The broken lines above and below the OoC indicate where the interscala bones were. The endolymph and the perilymph are separated by the OoC. The red lines indicate OCT beam path, whose angular range (θ_OCT_) was limited by the modiolus and the lateral wall. ***E***, ***F***, Reliable identification of different OoC structures was critical for this study. The diagram in ***F*** corresponds to the B-scan image in ***E***. TM: tectorial membrane, RL, reticular lamina; IHC, inner hair cell; OHCs, outer hair cells; HB, hair bundle; IPC, inner pillar cell; OPC, outer pillar cell; BM, basilar membrane.

Thirty-six young Mongolian gerbils (15–30 d old, either sex) were used for the experiments according to the institutional guidelines of the University Committee on Animal Resources at the University of Rochester. From deeply anesthetized gerbils using isoflurane, the cochlea was isolated and placed in a Petri dish, filled with a solution containing the following (mm): 145 Na-Gluconate, 7 NaCl, 3 KCl, 5 NaH_2_PO_4_, 0.1 MgCl_2_, 5 D-Glucose, 0.1 CaCl_2_, and 5 HEPES, pH 7.35 ± 3 and 300 ± 3 mOsm. The 1 mm section centered between 6.5 and 9.5 mm from the basal end was our target for measurement. After isolation, the basal and apical turns of the cochlea were removed with forceps and sharp blades leaving roughly one complete turn. The openings at the apical and basal end of the remaining tissue were sealed using cyanoacrylate glue (Fig. 1*A*, orange area). The reduced cochlear turn was transferred to a custom-designed microfluidic chamber that contained a perilymph-like solution. Glue was also applied along the circumference to fixate the excised cochlea on the chamber. Typical preparation time ranged from 60 to 80 min.

The distance from the basal end of the gerbil cochlea was indicated in millimeters. The full length of the gerbil cochlea was considered 12 mm. Our most measured location was *x* = 8.5 mm, where the expected BF is 1–1.5 kHz. The target location was determined by the relative location from a prominent anatomic structure—the bony protrusion between the oval and round windows. The most-measured location was one and a half turns away from the bony protrusion toward the apex, or one and one-eighth turns from the apical end. The 3-D coordinates of the medial and lateral ends of the basilar membrane in the literature (Plassmann et al., 1987) were used to translate the number of turns into the distance in mm. For instance, a quarter turn basal or apical from the most-measured location corresponded to *x* = 9.5 and *x* = 7.5 mm, respectively.

### Microfluidic chamber

The microfluidic chamber system, fabricated using stereolithography, was designed to separate the two fluid spaces across the cochlear epithelium. By doing so, mechanical, chemical, and electrical potentials could be applied across the cochlear epithelium. The mechanical stimulation was delivered through an opening sealed with elastomer, which was in contact with a piezoelectric actuator (Fig. 1*B*, red arrow). The other opening of the bottom channel served as a pressure release (analogous to the function of the round window in the natural cochlea). A pair of inlet-outlet ports were used to refresh the fluid in the bottom chamber. After placing and sealing the reduced cochlear turn on the chamber slit, the top fluid was replaced with an endolymph-like solution containing the following (in mm): 145 KCl, 0.1 CaCl_2_, 4 HEDTA, 10 K-HEPES, 8 Glucose, and 2 Na-Pyruvate, pH 7.35 ± 3 and 300 ± 3 mOsm.

### Viability of tissue

Two morphologic features were observed to judge the status of each preparation. First, the tectorial membrane attachment to the OoC was vulnerable to surgical or chemical agitations. Thus, a secure attachment reflected structural integrity of a preparation. Second, the morphology of OoC cells was sensitive to incomplete separation between the two fluid compartments. In rare occasions of incomplete separation, the cells in the OoC swelled appreciably within a few minutes of endolymph application.

### Experimental design and statistical analysis

#### Stimulation and measurements

Mechanical stimulations were applied using a piezoelectric actuator (PC4WM, Thorlabs) driven by a high-voltage amplifier (E-505, Physik Instrumente). The piezoelectric actuator tip vibrated 60 nm for 1 V driving voltage at 1 kHz. A typical driving voltage of 0.3 V resulted in ∼1 Pa of pressure near the slit at 1 kHz. To measure the pressures applied to the tissue, a hydrophone was installed at the location at which the distance from the stimulating port was comparable to the tissue. (Fig. 1*B*). The hydrophone was made from an electret microphone by coating its top surface with a thin layer of epoxy glue for waterproofing. The hydrophone was calibrated by measuring the vibrations of the artificial membrane (in place of the cochlear tissue), the compliance of which was known. The hydrophone was stable over months, but its operating frequency range was narrow (0.3–5 kHz). Stimulus functions were generated using MATLAB codes that controlled a data acquisition board (PCI-6353, National Instruments). A commercial OCT system (Ganymede, Thorlabs) was used for displacement measurements. The system uses a light source with 900 nm center wavelength, and its scanning unit has an A-scan rate up to 100 kHz. The system was modified to use a 20× objective (NA, 0.4; Mitutoyo) to enhance optical resolution.

#### Static displacement analysis

In our standard (dynamics) protocol, there were four fluid surfaces open to ambient air—the top chamber, pressure release, and the surfaces of inlet and outlet fluid vessels. Before placing the reduced cochlea, their water levels were in equilibrium so that there would be no pressure difference across the slit. After placing and sealing a cochlear turn, the pressure release and inlet openings were closed using a valve leaving only the fluid inlet that was connected to a fluid vessel (Fig. 1*A*). The inlet vessel water column level was adjusted by a connected syringe. A 0.1 ml volume change resulted in a 0.57 mm change in the water column height, which was equivalent to 5.6 Pa pressure change. As the pressure step either increased or decreased every 20 s, B-scan images of the OoC were taken. From image correlations between consecutive images using the MATLAB normcorr2 function, the displacement vector of each image pixel was obtained. This image analysis method was verified by analyzing a pair of sample images with known displacement. The error in estimating 100 nm displacement along the optical (transverse) axis was ∼10%. The level of pressure step (5.6 Pa) resulted in displacement between 200 and 1200 nm, depending on measurement location.

#### Two-dimensional vibration analysis

The measurement angle was defined by the angle of basilar membrane orientation. A few dozen M-scans were acquired across an OoC radial section, 2–3 µm apart (hereafter referred to as a “spatial sweep”). To get the 2-D motion of an OoC section, spatial sweeps were performed at two angles. The measurement angle was adjusted by rotating the optical head of the OCT system with respect to the microchamber. The light path obstructed by the lateral wall or the modiolus (Fig. 1*D*) limited the angular range between 30 and 50°. The stimulating frequency for spatial sweep experiments was near or below the response peak, which was near 1 and 2 kHz for 8.5 and 7.5 mm from the basal end, respectively. Modest frequency change (<0.25 octaves) near those frequencies resulted in little change in vibration patterns (Jabeen et al., 2020). The radial and transverse components $d_{r}$ and $d_{t}$ of 2-D vibration were calculated from the displacement at two angles as follows:

(1) $$\left[\begin{array}{c} d_{r}\\ d_{t} \end{array}\right]={\left[\begin{array}{cc} \text{sin}\theta_{1} & \text{cos}\theta_{1}\\ \text{sin}\theta_{2} & \text{cos}\theta_{2} \end{array}\right]}^{-1}\left[\begin{array}{c} d_{1}\\ d_{2} \end{array}\right],$$

where $d_{1}$ and $d_{2}$ are the displacement in complex number (having both amplitude and phase information) at the orientation angle of $\theta_{1}$ and $\theta_{2}$, respectively.

In presenting the experimental data, *N* represents the number of cochleas (animal), and *n* represents the number of measurements.

#### Computational modeling

**Figure 2.**
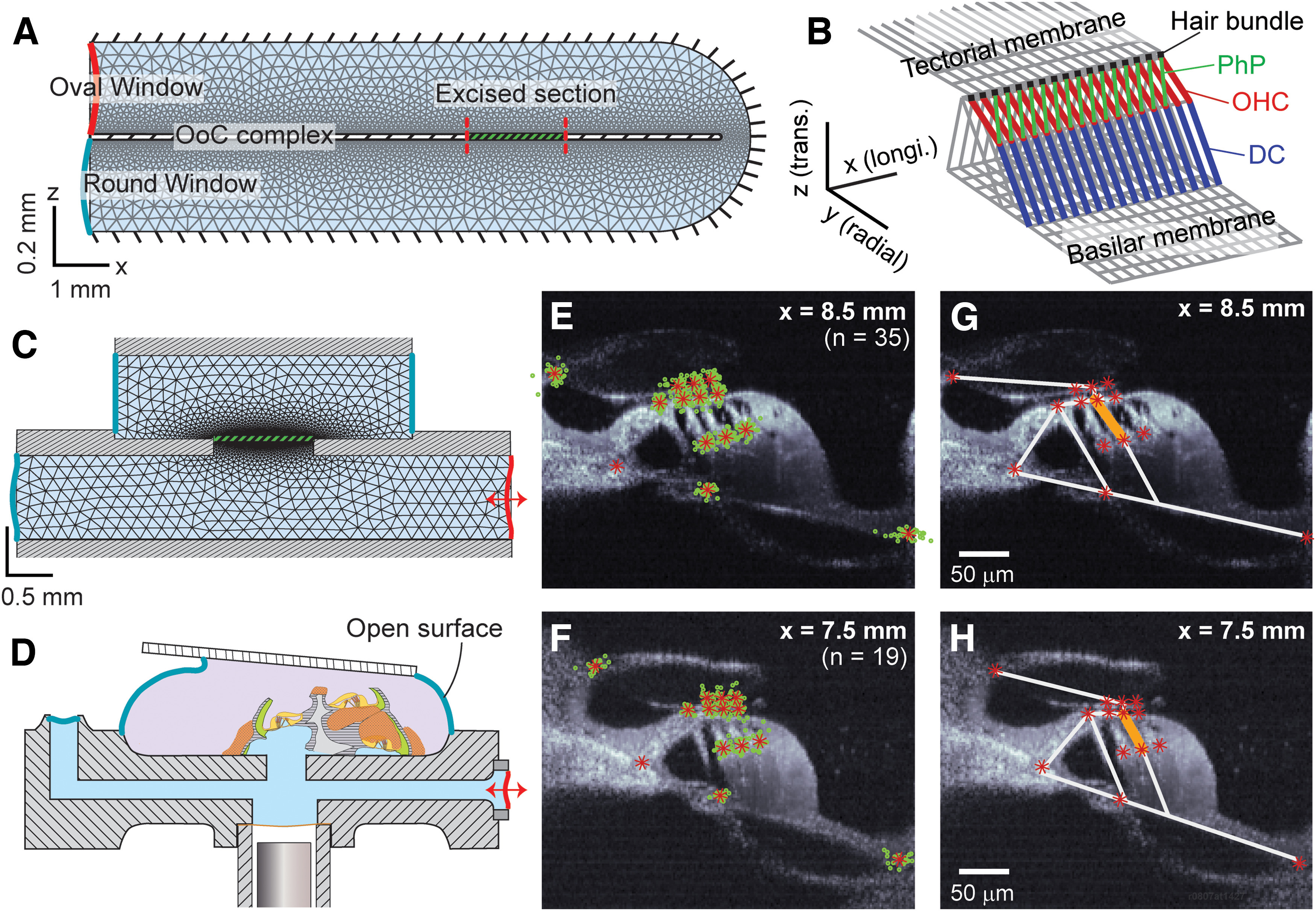
Computational model of excised cochlea based on measured geometry. ***A***, FE model of the full cochlea. ***B***, A piece of the OoC FE model (span of 0.2 mm is shown). The OHCs, PhP, and DCs are distinguished by different colors. ***C***, FE model of the microfluidic chamber. The thick broken line separating the top and bottom fluid spaces represents the excised cochlear section. The mesh grid is finer near the cochlear section to match the structural grid size. ***D***, Microchamber diagram was redrawn here to illustrate the fluid boundaries corresponding to the FE model in ***C***. Diagram is not to scale. ***E***, ***F***, To determine the geometry of FE models, 14 anatomic landmarks (root of the inner pillar cell, tip of the tunnel of Corti, root of the outer pillar cell, top and bottom of three OHCs, tips of the HBs, lateral end of the BM and medial edge of the TM) were collected from 35 and 19 preparations, whose longitudinal locations are 8.5 and 7.5 mm from the basal end, respectively. The green circles are from individual cochleae, and the red asterisks are mean positions. ***G***, ***H***, FE models (white lines) of the radial sections are shown together with the measured anatomic points.

A computational model of an excised cochlea in a microchamber was modified from an existing full cochlear model (Fig. 2*A*,*B*; Liu et al., 2015, 2017; Zhou and Nam, 2019). All governing equations (thus the matrices of the numerical model) remained the same as the previous studies. Changes were limited to the geometry and the boundary conditions of fluid domain to represent the reduced cochlea in a microfluidic chamber. This implies that findings of this study can readily be extended to the whole cochlea simulations, without adjusting model parameters or governing equations.

The model incorporated fluid dynamics and micromechanics of the OoC. The FE method was used in both fluid and solid domains to solve for fluid pressures and structural displacements (Fig. 2*A*).

The governing equation for hydrodynamics came from the Navier–Stokes equation and the continuity equation. Assuming the fluid is incompressible and inviscid, the Navier–Stokes equation was reduced to the following:

(2) $$\nabla^{2}p=0,$$

where $\nabla^{2}$ is the Laplacian operator and p is the pressure in the fluid space. The boundaries of the fluid domain were as follows. Two fluid structure boundaries were considered; the top and bottom fluid interaction surfaces were represented by the tectorial and basilar membranes, respectively. At those boundaries, the pressure gradient normal to the surface was proportional to the accelerations of the surfaces, or dp/dz=−ρa, where ρ is the fluid mass density. The fluid forces f_FLD_ on the tectorial membrane and the basilar membrane were calculated as the pressure difference across the interfaces multiplied by the effective interacting surface areas. The governing equations for the fluid domain were discretized and expressed in the matrix form as follows:

(3) $$A_{pp}p+A_{pa}a=b.$$

$A_{pp}$ corresponds to the Laplace operator. $A_{pa}$ represents the boundary conditions at the OoC complex fluid interacting surfaces, a is the acceleration vector of the OoC complex interacting nodes, and b represents the prescribed pressure boundary conditions including the pressure input and release ports.

OoC mechanics was represented by a system consisting of mass (M), damping (C), and stiffness (K) matrices subjected to a fluid force ($f_{FLD}$). For C, the Rayleigh damping was used. The following global equation:

(4) $$M\ddot{x}+C\dot{x}+Kx=f_{FLD}$$

was used to solve for the displacement vector (x); $f_{FLD}$ was determined by the pressure input at the stapes and a matrix $A_{ap}$ relating pressure to forces freedom at the interacting surfaces as follows:

(5) $$f_{FLD}=A_{ap}p.$$

Since no time derivatives of the pressure appear in the governing equations, the pressure was substituted using Equation 3 as follows:

(6) $$f_{FLD}=A_{ap}A_{pp}^{-1}\left(b-A_{pa}a\right).$$

Thus, the combined governing equation for the fluid and structure domains is the following:

(7) $$M_{EFF}\ddot{x}+C\dot{x}+Kx=f_{EFF},$$

where $M_{EFF}$ represents the overall inertia consisting of structural mass and effective fluid mass, and $f_{EFF}$ is the effective load because of the stapes motion in the following:

(8) $$M_{EFF}=M+A_{ap}A_{pp}^{-1}A_{pa}$$

(9) $$f_{EFF}=A_{ap}A_{pp}^{-1}b.$$

The governing equations were then solved in the frequency domain.

The fluid domain was represented by two rectangular fluid spaces separated in the middle by a partition (Fig. 2*C*). A slit was in the middle of the separating partition, which was sealed by the OoC. For the lower fluid compartment, mechanical input was through the left end, where the pressure was given. The pressure release port was located at the right end. For the upper compartment, fixed boundary conditions on top represent the coverslip fixed to the objective lens. The left and right boundaries of the top chamber were open to ambient air. The mesh grid was finer around the OoC to obtain more accurate results while saving computational cost.

The OoC FE model comparable to the reduced cochlear turn (4 mm) was created (Fig. 2*B*, only a 0.2 mm piece is shown). The displacements of both the left and right (apical and basal) ends were fixed. A radial section of the OoC FE model had 23 nodes (13 at the basilar membrane, 6 at the tectorial membrane, and 5 in the OoC). The lines (elements) in Figure 2*B* represent the connectivity between nodes, and the cross-sectional area of elements represents the proper mass of the OoC (e.g., the thickness of the tectorial and basilar membranes). The longitudinal gradients of geometry and mechanical properties were considered in the model.

Physiologically accurate geometry is critical for the purpose of this study because this study compared measured and simulated vibrating direction as well as amplitude. Our OCT images provided detailed geometric information of OoC fine structures at which vibrations were measured (Fig. 2*E–H*). Figure 2, *E* and *F*, shows the anatomy at two locations. From image pairs acquired at different orientations, the refractive index of the OoC tissue was obtained to be 1.40. Figure 2, *G* and *H*, compares the geometry of models (white lines) and OCT images (asterisks). The width of the basilar membrane was 299 ± 12 µm (mean ± SD, *n* = 35 images of *N* = 28 cochleas) at *x* = 8.5 mm and 274 ± 9 µm (*n* = 19, *N* = 11) at *x* = 7.5 mm.

### Data availability

The OCT data and the FE analysis code used in this study are available at https://figshare.com/search?q=10.6084%2Fm9.figshare.20098112.

## Results

**Figure 3.**
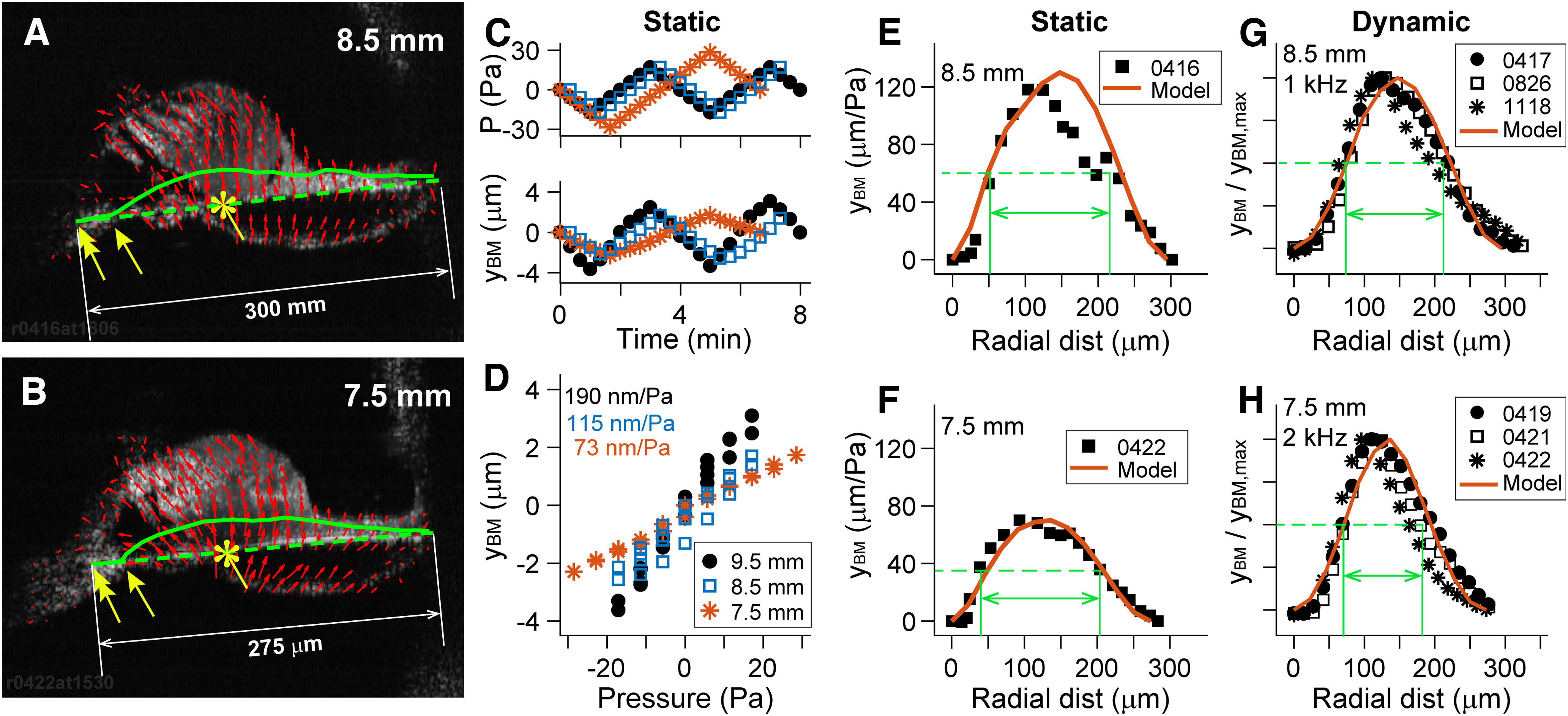
Volume compliance of the OoC complex. ***A–G***, The OoC deformations because of hydrostatic (***A–F***) and hydrodynamic stimulations (***F***, ***G***) were measured. ***A***, ***B***, Displacement vector field (red arrows). The asterisks indicate the root of the first-row Deiters cell, where the peak displacement occurs. Double-head yellow arrows show roots of the inner hair cells, and single-headed yellow arrows show onset positions of BM deformation. Results from two locations are shown (8.5 and 7.5 mm from the basal end). Volume compliances were obtained by computing the area between the deformed and undeformed basilar membrane (solid and broken green lines). ***C***, Pressure level increased or decreased by 5.6 Pa every 20 s, and B-scan images were acquired at each step. Peak basilar membrane displacement (y_BM_) over time. ***D***, Displacement–pressure relationship of three samples representing the locations of 9.5, 8.5, and 7.5 mm. ***E***, ***F***, Basilar membrane deformation because of hydrostatic pressure at two locations. ***G***, ***H***, Basilar membrane deformation because of hydrodynamic pressure at two locations. The green arrows indicate full-width at half-maximum (***E–H***). All data in this plot are measurements, except the red solid curves in ***E–H***, which are from computer model simulations.

**Figure 4.**
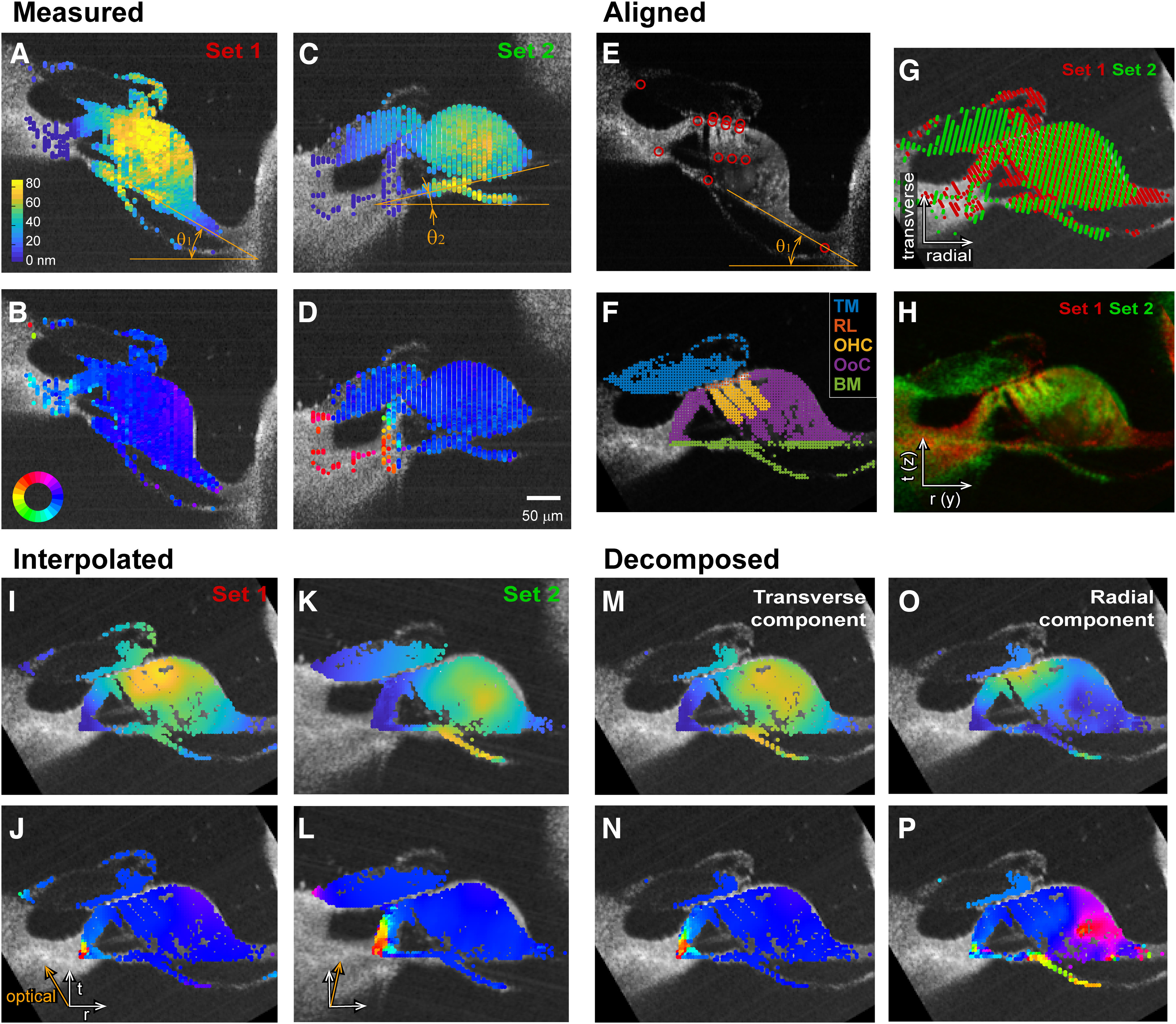
Two-dimensional motion analysis. Through four stages, 2-D motion was obtained from two sets of OCT measurement. ***A–D***, Two sets of vibration measurement at different orientations. In this example θ_1_ = −30° and θ_2_ = 13° for Sets 1 and 2, respectively. Top (***A***, ***C***) and bottom (***B***, ***D***) represent the vibration amplitude and phase, respectively. The scales of amplitude and phase remain the same in other places in this figure. The stimulating frequency was 800 Hz. ***E–H***, The two datasets were aligned to each other by rotating and translating. Fourteen anatomic points were identified (***E***). Different structures were defined based on the anatomic points (***F***). The image pixels with a sufficient number of nearby OCT data points and good imaging signal strength were indicated. Alignment of the two datasets (***G***). Sets 1 and 2 images are shown in corresponding color layers (***H***). ***I–L***, Scattered data points were interpolated. The optical axis for each set is indicated with orange arrows (***J***, ***L***, bottom left corner). ***M–P***, Transverse and radial components of vibrations were obtained from ***I–L*** using Equation 1.

**Figure 5.**
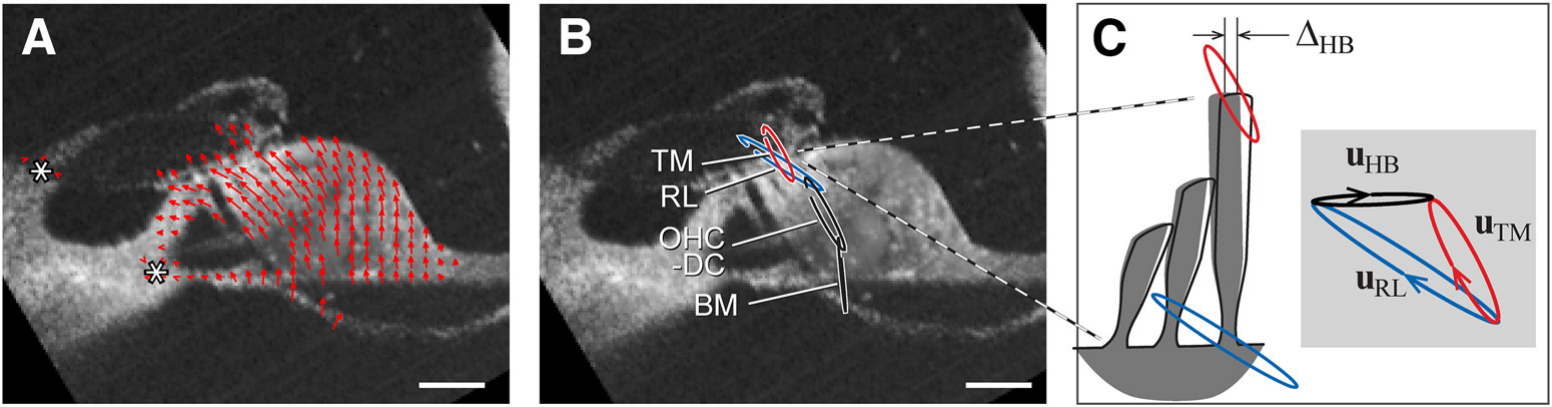
Measured motion of OoC structures. ***A***, Vector plot of Figure 4*M–P* indicating the direction and amplitude of motion across the OoC complex because of pure tone simulation. ***B***, Motion at four anatomic points were used for analyses. (***C***) Hair bundle deflection, Δ_HB_ = |**u**_HB_|, was obtained from the difference between TM and RL motion vectors (**u**_HB_ = **u**_TM_ – **u**_RL_). Scale bars: 50 µm.

**Figure 6.**
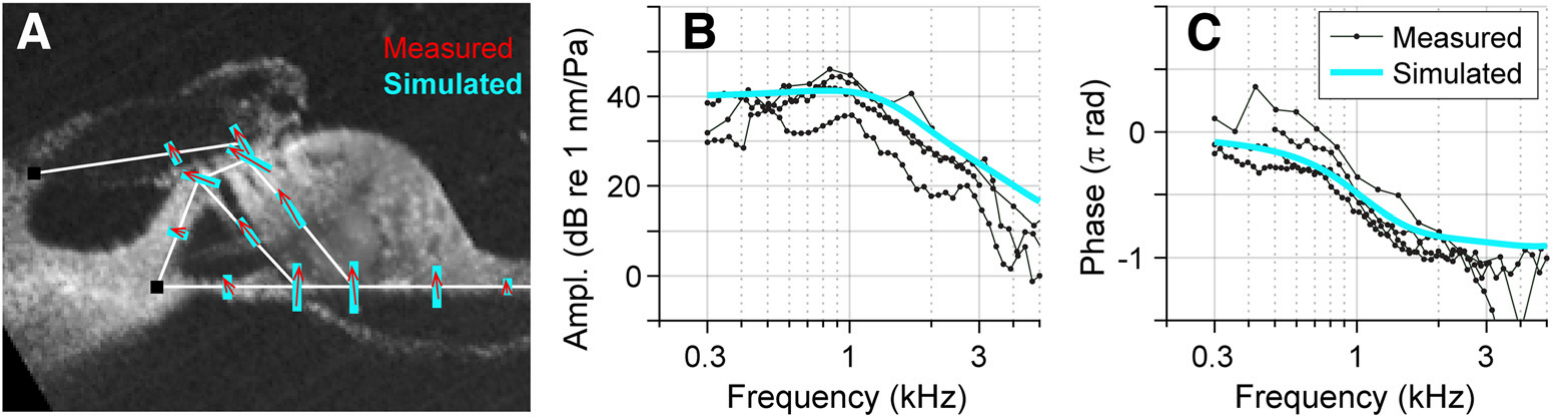
Comparing simulations and measurements. ***A***, Vibration pattern shown as a vector field. Simulated results (thick light-blue bars) shown together with measured results (thin red arrows). ***B***, ***C***, Vibration amplitude and phase at the basilar membrane when subjected to sinusoidal pressures. Three sets of measured data (thin curves with markers) are presented together with simulated results (thick curves). Measurements and simulations were performed at the nominal location of *x* = 8.5 mm.

**Figure 7.**
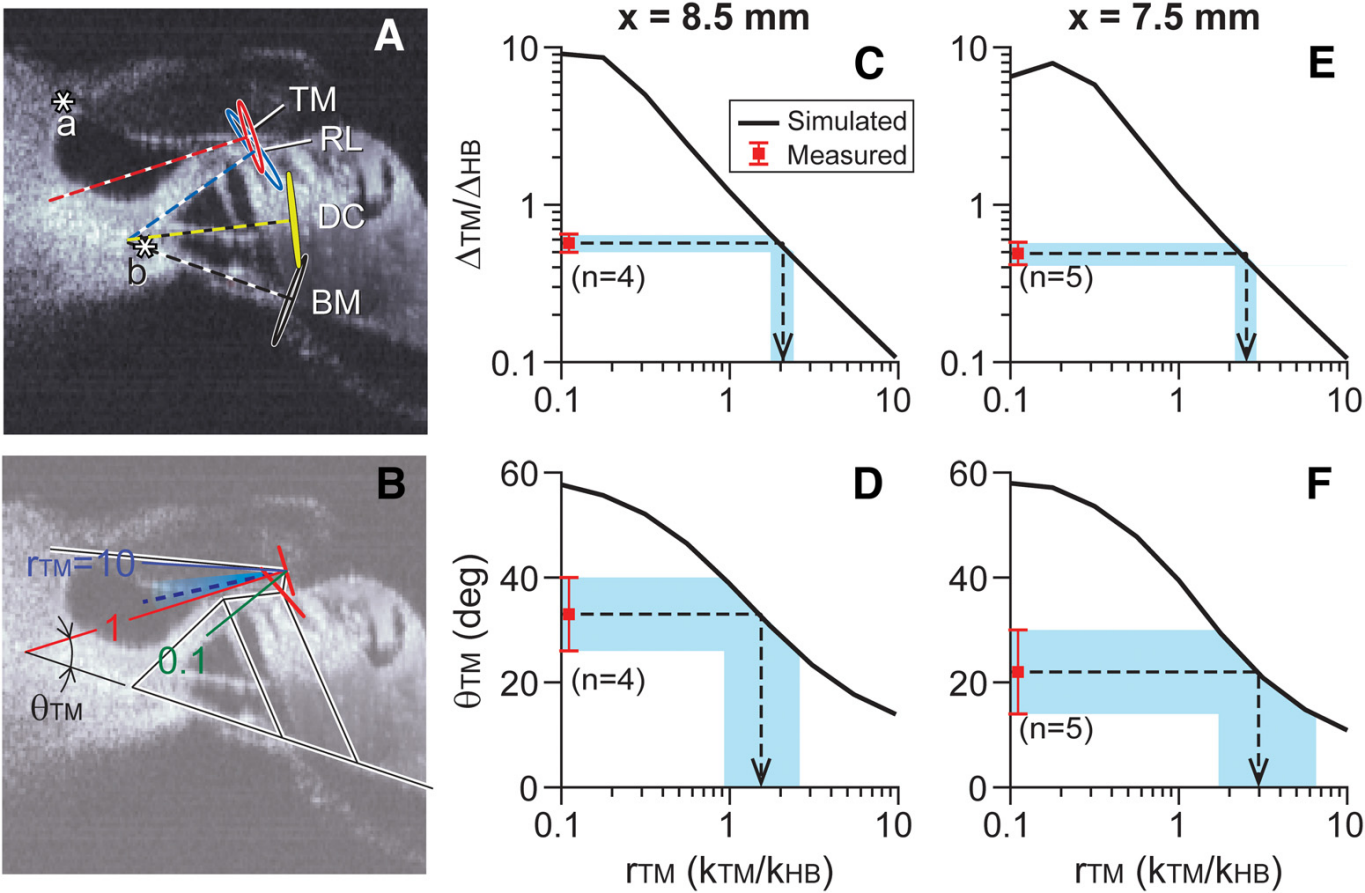
Tectorial membrane stiffness. ***A***, Measured data. Oscillatory motion at 1 kHz was drawn at four anatomic points, the TM, RL, DC, and BM. Although the OoC structures rotate roughly about the inner pillar cell root at b, the tectorial membrane does not rotate about its physical attachment at a but rotates between a and b. ***B***, Simulated results shown together with measured data. The direction of TM motion (θ_TM_) depends on the stiffness ratio between the tectorial membrane and the hair bundle *r*_TM_ (*k*_TM_/*k*_HB_). The broken line and the shaded area indicate measured mean and SD of θ_TM_. ***C–F***, FE model simulation shown together with measurements. The square symbol with error bounds next to the vertical axes represents the mean and SD of measurements. The solid curves are simulated results. The horizontal axis represents simulated *r*_TM_ (*k*_TM_/*k*_HB_). Results from two longitudinal locations (*x* = 8.5 and 7.5 mm) are presented.

**Figure 8.**
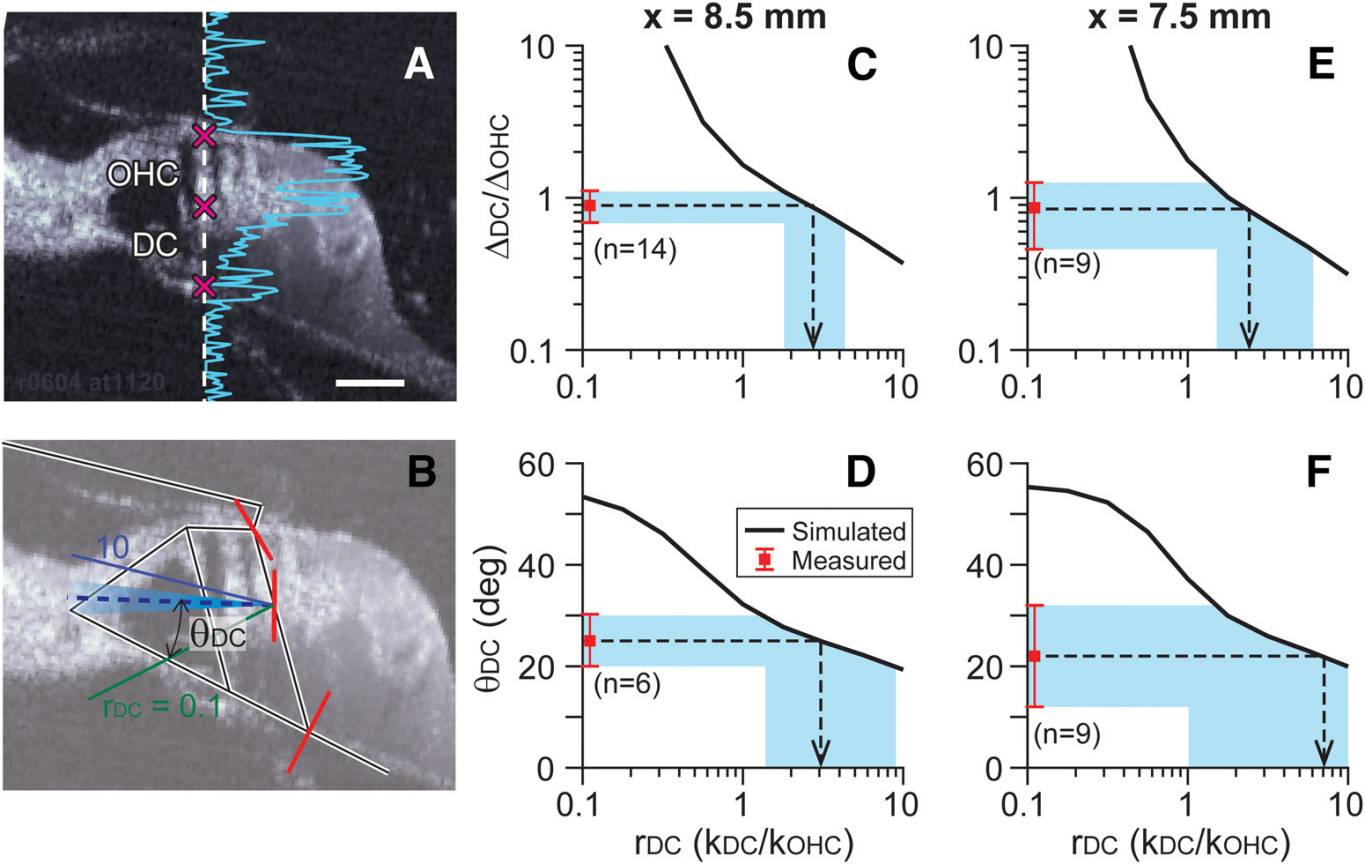
Deiters cell stiffness. ***A***, Measured data. The OCT beam path (vertical broken line) was aligned with the outer hair cell length. The blue curve indicates the strength of optical signal. From the displacements at three points (cross marks), the deformation ratio between Deiters cell and outer hair cell (Δ_DC_/Δ_OHC_) was obtained. ***B***, Simulated results (solid lines) shown together with measured data (broken line). The movement direction of Deiters cell outer hair cell joint. Two solid lines (blue and green) are simulated results with *r*_DC_ = 0.1 and 10, where *r*_DC_ = *k*_DC_/*k*_OHC_. Broken line and shaded area indicate measured mean and SD of the movement direction. ***C–F***, FE model simulation shown together with measurements. The square symbols with error bands next to the vertical axes represent the mean and SD of measurements. The solid curves are simulated results. The horizontal axis represents simulated *r*_DC_ (*k*_TM_/*k*_HB_). Results from two longitudinal locations (*x* = 8.5 and 7.5 mm) are presented.

**Figure 9.**
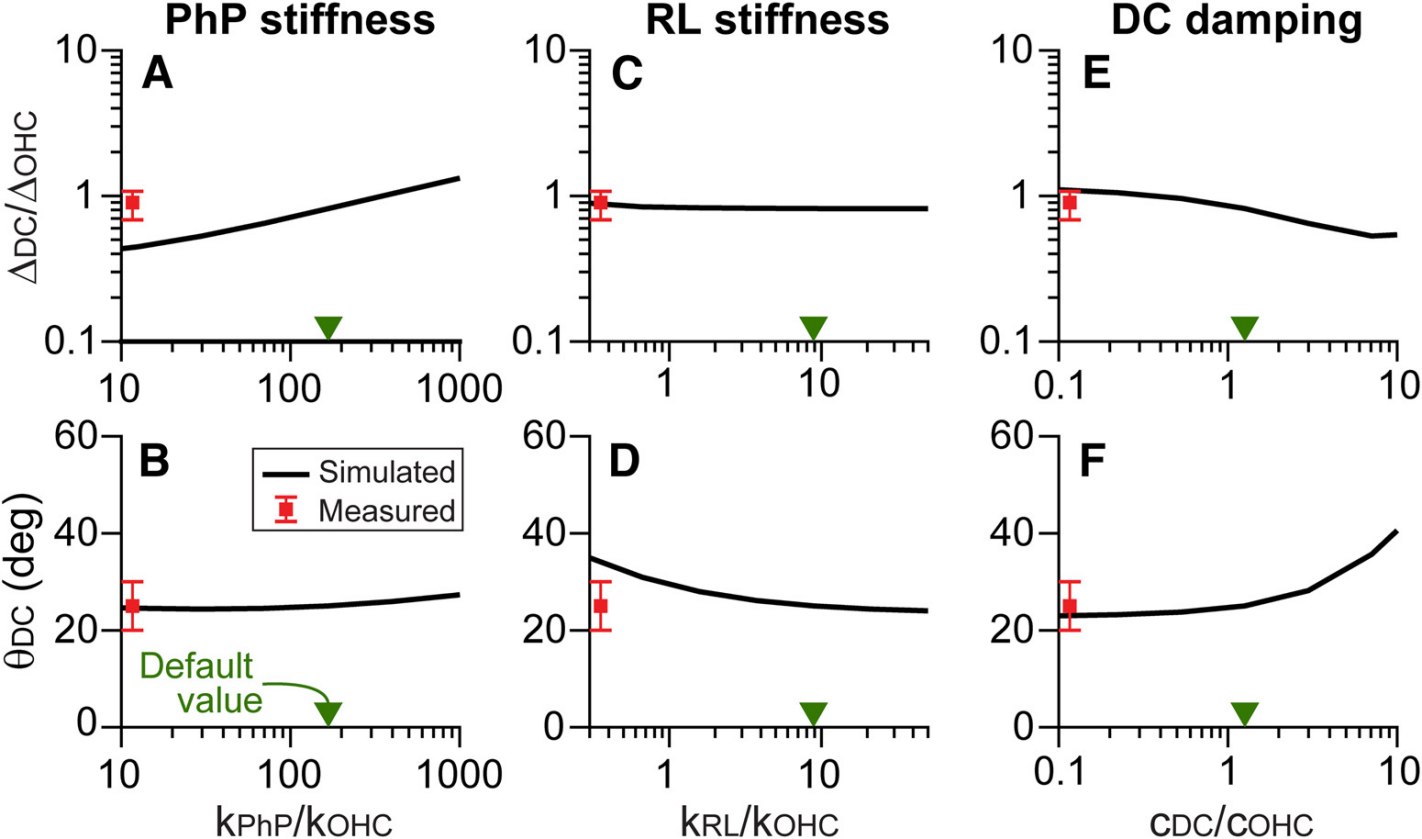
Other properties affecting the motion of outer hair cell/Deiters cell joint. ***A–F***, The FE model simulated the motion of the joint between outer hair cell and Deiters cell at *x* = 8.5 mm, represented by Δ_DC_/Δ_OHC_ and θ_DC_, depending on the stiffness of Deiters cell phalangeal process (*k*_PhP_, ***A***, ***B***), the stiffness of reticular lamina (*k*_RL_, ***C***, ***D***), and the damping of Deiters cell (*c*_DC_, ***E***, ***F***). The square symbols with error bands next to the vertical axes represent the mean and SD of measurements (the same as those in Fig. 8*C*,*D*). The triangular symbols (▾) on the horizontal axis indicate the default property used in the FE model.

Experimental results of two protocols were plotted together with relevant computer model simulations. In one set of experiments (Fig. 3*A–F*), excised cochlear tissues were subjected to hydrostatic pressures. In the other set of experiments (Fig. 3*G*,*H* and Figs. 4–9), cochlear tissues were subjected to hydrodynamic pressures, and resulting vibrations were measured at two orientation angles. The fully deformable 3-D FE model was validated by comparing simulated static and dynamic results with relevant measurements (Fig. 3*E–H*; see Fig. 6). While here Figures 4 and 5 present measured data only, all other figures copresent measure and simulated results. In Figures 7–9, vibration amplitude and direction measured at specific anatomic points were compared with hydrodynamic model simulations to determine mechanical properties of the tectorial membrane, Deiters cell, and reticular lamina.

### Static deformation and volume compliance of the OoC complex

OoC deformation due to hydrostatic pressure was measured. Nearly all modern data regarding the OoC complex stiffness (with the exception of our previous study Marnell et al., 2018) were from contact (Hertzian) stiffness measurements, which required careful postanalysis to derive functionally relevant volume compliance. Our two fluid space chamber system and high-resolution imaging enabled us to measure the volume compliance of OoC complex explicitly (Fig. 3). Unlike other experiments in this article, the scala tympani faced upward in these measurements to visualize the basilar membrane better.

Our observation regarding the radial deforming pattern of the basilar membrane was consistent with the previous observations by Cooper et al. (2018), despite different measurement locations (Cooper, 2000; Cooper and van der Heijden, 2018). For example, the peak displacement of the basilar membrane occurred near the root of the first-row Deiters cells (Fig. 3*A*,*B*, asterisks). The basilar membrane was deflected as if it were clamped at the root of the inner pillar cell (double-headed arrow), and as if it were a pin joint at the tip of the inner spiral lamina (single-headed arrow). The pressure-displacement relationship was highly linear (*R*^2^ > 0.9; Fig. 3*D*). For the presented samples in Figure 3, the compliance values were 190, 115, and 70 nm/Pa at *x* = 9.5, 8.5, and 7.5 mm, respectively (peak basilar membrane displacement divided by applied pressure). The volume compliance was obtained from the displaced area of the basilar membrane (Fig. 3*A*,*B*, area between solid and broken lines). The volume compliance values were 28.2, 16.1, and 10.4 mm^4^/N at *x* = 9.5, 8.5, and 7.5 mm. In two previous measurements of the gerbil cochlear stiffness, one study reported a 2.7-fold change per millimeter in the cochlear length (Emadi et al., 2004), and the other study reported 1.4-fold change per millimeter (Naidu and Mountain, 1998). Using our OoC FE model, contact stiffness values of the literature in N/m can be compared with pressure compliance in nm/Pa because the continuum mechanics model can simulate both concentrated and distributed forces. Our measured compliance values correspond to contact stiffness values of 44, 71 and 120 mN/m at *x* = 9.5, 8.5, and 7.5 mm, respectively. At *x* = 7–8 mm of the gerbil cochlea, Emadi et al. (2004) obtained 40–80 mN/m, and Naidu and Mountain (1998) reported 300–800 mN/m. To summarize, the measured results could be reproduced by independent computer model and are comparable to existing data.

The basilar membrane deforming pattern because of dynamic stimulations was simulated and measured (Fig. 3*G*,*H*). To obtain a similar pattern, the FE model needed clamped boundary conditions (no translational or rotational displacement) at the medial and lateral edges of the basilar membrane. The primary parameters that determine the compliance of the OoC complex were the basilar membrane dimensions (thickness and width) and elastic moduli. The width was given based on our measurement, and the thickness of collagen fiber layers were based on the literature (Table 1). Given Young's modulus of the collagen fiber layers of 2 GPa, the displacement of the computer model compared reasonably well with measurements (Fig. 3*G*,*H*).

**Table 1.** Mechanical properties of the organ of Corti components of the gerbil cochlea

Structure	Parameter	*x* = 10 mm	*x* = 2 mm
Basilar membrane†	Width	330	180
	Fiber layer thickness	0.7	3.2
	Midpectinate thickness	55	35
	Young's modulus (*x*, *z*)	2000, 0.4	2000, 0.4
Outer hair cell soma	Diameter	7	9
	Length	56	24
	Young's modulus	0.005	0.005
Outer hair cell hair bundle	Height	10	4
	stiffness	3	40
Pillar cell	Diameter	4	6
	Young's modulus	400	400
Deiters cell (base, process)	Diameter	10, 1	10, 1.5
	Young's modulus	0.01, 40	0.01, 40
Reticular lamina (tunnel of Corti, OHC)	Thickness	5, 2	5, 2
	Young's modulus (*x*, *z*)	500, 0.2	500, 0.2
Tectorial membrane (root, body)‡	Width	156	110
	Thickness	25, 50	20, 30
	Young's modulus (x)	0.018, 0.0044	0.29, 0.074
	Young's modulus (z)	0.002	0.002

All dimensions are in μm (except the longitudinal distance in mm). Young's moduli are in MPa (10^6^ N/m^2^), and stiffness values are in mN/m (10^−3^ N/m). All properties were considered to vary exponentially along the distance, or $p=\text{exp}\left(s\left(x-x_{b}\right)-p_{b}\right),$ where $s=\left(\text{ln}(p_{b})-\text{ln}(p_{a})\right)/\left(x_{b}-x_{a}\right),$ and x is the distance in mm, and p is physical quantity. The subscripts a and b represent the two locations where the properties are defined (10 and 2 mm from the base).

†The basilar membrane is divided into two parts. The arcuate zone beneath the tunnel of Corti is considered having a half-thick fiber layer compared with the pectinate zone. The Young's modulus values are for the fiber layers (the tympanic layer was considered negligible in stiffness).

‡A radial section of the tectorial membrane is divided into two regions, the root attached to the spiral limbus and the body overlying the hair cell stereocilia, the root region being half long and half thick as the body.

The deforming pattern was narrower when dynamically stimulated than statically stimulated. The span of deforming pattern at the half-maximal level (the green arrows) was greater than the half-width of basilar membrane (0.59 times the width for Fig. 3*E* and *F*) when statically deformed, but it was less than the half-width (0.45 and 0.41 times the width for Fig. 3*G* and *H*) when dynamically deformed. The trend was consistent with model simulations (solid red curves).

### Two-dimensional vibrations of the OoC

Figure 4 presents our 2-D analysis procedure step by step. Forty to 50 M-scans across the span of OoC were collected at two orientation angles while the preparation was subjected to pure-tone stimulation (0.7–1 kHz for *x* = 8.5 mm; 1.5–2 kHz for *x* = 7.5 mm). In this example case, the basilar membrane was tilted by −30 and 13 degrees from the horizontal line. The image resolution (0.7 and 1.9 µm along the lateral and optical axes, respectively) was sufficient to resolve individual outer hair cells but insufficient to distinguish hair bundles. Essentially, the entire OoC vibrated in phase (Fig. 4*B*,*D*), indicative of passive mechanics (insensitive cochlea). Measurements at two orientations were aligned to each other after identifying anatomic points including the ends of three rows of outer hair cells and the boundary of tectorial membrane, basilar membrane, and lateral compartment (Fig. 4*E*,*F*). Figure 4, *G* and *H*, shows aligned data points and overlapped B-scan images. The optical reflectivity of the tectorial membrane was dependent on the angle of the laser beam. When the lateral end of the tectorial membrane was tilted downward, only the periphery was sufficiently reflective (Fig. 4*A*,G,*H*, Set 1), whereas the entire tectorial membrane was reflective when it was tilted upward (Fig. 4*C*,G,*H*, Set 2). With respect to the rectilinear grid points in the OoC complex (Fig. 4*F*), complex amplitudes of vibrations were interpolated from nearby measured values. The aligned and interpolated results are shown Figure 4, *I–L*. Using Equation 1, the vibrations along the respective optical axes were decomposed into the transverse (Fig. 4*M*,*N*) and the radial (Fig. 4*O*,*P*) components. The decomposed plots demonstrate how transverse motion of the basilar membrane turns into deflection of hair bundle, consistent with classical OoC kinematics. For example, the basilar membrane vibrated mostly transversely. The reticular lamina and the tectorial membrane moved similarly in the transverse direction, but the radial motion was much greater in the reticular lamina, which resulted in hair bundle deflection. Although the raw data showed little variation in phase within the OoC (Fig. 4*B*,*D*), the decomposed radial motion showed a large variance in phase (Fig. 4*P*).

The overall displacement vector field (Fig. 5*A*) looked reasonable in that the tectorial membrane and the OoC rotate approximately about their respective lateral attachment points (Fig. 5*A*, spiral limbus and spiral lamina marked by the asterisks). The motion at any point was elliptical (Fig. 5*B*), reflecting nonperfect matching of phase between the measurements at two orientations. The vectors represent the major axis of the elliptical motion. Hair bundle deflection was obtained from the relative motion between the tectorial membrane and the reticular lamina (Fig. 5*B*,*C*), similar to Lee et al. (2016). The displacement vectors (amplitudes and directions) measured at substructures of the OoC complex, when combined with detailed model simulations, can be exploited to identify the functionally relevant stiffness of two key structures—the tectorial membrane and the Deiters cell. Roughly speaking, the tectorial membrane is mechanically in series with the hair bundle where mechanical vibrations turn into electrical signals. The Deiters cell is mechanically in series with the outer hair cell of which electromotility underlies cochlear amplification. For further analysis we focused on four anatomic points (Fig. 5*B*). They are the tectorial membrane and the reticular lamina at the second-row outer hair cell bundle, the joint between the outer hair cell and the Deiters cell and the root of Deiters cell, which correspond to TM, RL, OHC-DC, and BM in Fig. 5*B*. The arrows in Figure 5*A* represent the major axis of elliptical motions.

In Figure 6, FE model responses to pure-tone stimulations were compared with measurements. Figure 6*A* presents the comparison in the spatial domain. On top of measured motions (red arrows), simulated motions (thick green bars) were plotted. Figure 6, *B* and *C*, presents the comparison in the frequency domain; vibration amplitude and phase measured at the middle of the basilar membrane were compared at frequencies between 0.3 and 5 kHz. As our preparation widely opened the scalae and reduced the cochlear coil, the excised OoC responded as a second-order resonator. At the location of *x* = 8.5 mm, the corner frequency was ∼1 kHz. The amplitude responses showed a slope of −12 dB/octave at frequencies greater than the corner frequency, and the phase decreased by 180° across the corner frequency.

As we confirmed that the model reasonably reproduces measured responses both in static (Fig. 3*E*,*F*) and dynamic (Fig. 3*G*,*H*, and Fig. 6) aspects, in the following sections, experimental observations are presented together with computational model analyses to identify the mechanical properties of the tectorial membrane and the Deiters cells.

### Tectorial membrane stiffness

The stiffness ratio between the tectorial membrane and the hair bundle was obtained from their deformation ratio. Specifically, the axial (radial) stiffness component of the tectorial membrane (*k*_TM_) and the bending stiffness component of the hair bundle, (*k*_HB_), were considered. When two elastic structures are in series, the force carried by the two components deform them according to their stiffness ratio. In the case of the tectorial membrane/hair bundle complex, *k*_TM_Δ_TM_ ≈ *k*_HB_Δ_HB_, where Δ_TM_ is the radial elongation of the tectorial membrane and Δ_HB_ is the deflection of the hair bundle.

From 2-D vibration measurements, we obtained the deformation ratio (Δ_TM_/Δ_HB_) of 0.57 ± 0.07 (mean ± SD, *N* = 4) at *x* = 8.5 mm, and 0.49 ± 0.08 (*N* = 5) at *x* = 7.5 mm (Fig. 7*C*,*E*, square symbols with error bounds). To estimate the stiffness ratio between the tectorial membrane and the hair bundle (*r*_TM_ = *k*_TM_/*k*_HB_), fully deformable 3-D FE model of the OoC complex (Fig. 2) was used. For *x* = 8.5 and 7.5 mm locations, OoC complex vibrations to 1 kHz and 2 kHz pure tones, respectively, were simulated. A series of simulations was performed with different axial stiffness values of the tectorial membrane, resulting in *r*_TM_ between 0.1 and 10. From the simulated results, the relationship between *r*_TM_ and Δ_TM_/Δ_HB_ was obtained (Fig. 7*C*,*E*, diagonally running curves). The value of *r*_TM_ corresponding to measured value of Δ_TM_/Δ_HB_ was identified as 2.1 ± 0.3 at *x* = 8.5 mm and 2.5 ± 0.3 at *x* = 7.5 mm (Fig. 7*C*,*E*, arrows). Although the simple 1-D equation is only an approximation of 3-D mechanics, 3-D model simulations were in line with the approximation. For example, the solid curves in Figure 7, *C* and *E*, showed the slope of −1 in the log-log plot, confirming the relationship of Δ_TM_/Δ_HB_ ≈ 1/(*k*_TM_/*k*_HB_).

As an alternative method to estimate *r*_TM_, the direction of tectorial membrane motion was analyzed. Two asymptotic cases can be considered. If the tectorial membrane is much stiffer than the hair bundle (*r*_TM_ *= k*_TM_/*k*_HB_ > 10), the tectorial membrane may move like a rigid body rotating about its attachment point to the spiral limbus (Fig. 7*A*, a). Conversely, if it is highly compliant (*r*_TM_ < 0.1), the tectorial membrane may move like a floating mass sitting on the OoC. In this latter case, the tectorial membrane will rotate about the root of the inner pillar cell (Fig. 7*A*, b) together with the OoC. If the tectorial membrane has a stiffness value comparable to hair bundle stiffness (0.1 < *r*_TM_ < 10), the tectorial membrane will move as if its apparent center of rotation is in between a and b.

Figure 7*A* shows measured displacement vectors obtained from a sample at *x* = 8.5 mm. The direction of tectorial membrane vibration was consistent with the case of comparable stiffness (0.1 < *r*_TM_ < 10). Figure 7*B* shows simulated results (colored solid lines) together with measured results (broken line and shaded area indicate the mean and SD). As expected, as *r*_TM_ changes from 10 to 0.1, the simulated center of rotation moves from a to b. Measured vibrating direction was θ_TM_ = 33 ± 7 at *x* = 8.5 mm and θ_TM_ = 22 ± 8 at *x* = 7.5 mm (Fig. 7*D*,*F*, square symbol with error bounds next to the vertical axes). When compared with the FE model simulations, an *r*_TM_ value of 1.5 (*x* = 8.5 mm) and 3 (*x* = 7.5 mm) corresponded with the angles.

To simulate *r*_TM_ change, different elastic modulus values of the tectorial membrane were used while all other parameters remained the same. Note that the change of *r*_TM_ affected the direction of tectorial membrane motion (Fig. 7*E*,*F*), but the motion of other parts such as the reticular lamina was hardly affected (<1° while changing *r*_TM_ between 0.1 and 10).

### Deiters cell stiffness

The deformation ratio between Deiters cell and outer hair cell (Δ_DC_/Δ_OHC_) was used to estimate the relative stiffness of a Deiters cell with respect to outer hair cell, *r*_DC_ = *k*_DC_/*k*_OHC_ (Fig. 8*C*,*E*). Because the two cells are nearly aligned along their length axes (Fig. 8*A*), instead of measuring 2-D vibrations, the optical orientation was chosen so that the beam axis was approximately aligned with the first-row outer hair cell and Deiters cell. From the displacements of three points (Fig. 8*A*, cross marks), Δ_DC_ and Δ_OHC_ were obtained. The measured deformation ratios were 0.89 ± 0.21 (*N* = 14) at *x* = 8.5 mm and 0.89 ± 0.20 (*N* = 9) at *x* = 7.5 mm (Fig. 8*C*,*E*, square symbols and shaded spans).

A series of simulations were performed similar to the tectorial membrane case. The value of Deiters cell axial stiffness was adjusted over two orders of magnitude, 0.1 < *r*_DC_ < 10 to obtain the relationship between Δ_DC_/Δ_OHC_ and *r*_DC_ (Fig. 8*C*,*E*, diagonally running curves). When the stiffness ratio was *r*_DC_ = 2.7 ± 0.7 at *x* = 8.5 mm and 2.4 ± 0.7 at *x* = 7.5 mm, the simulated results were aligned with the experiment.

The movement direction of the outer hair cell/Deiters cell joint was not as informative as the deformation ratio in estimating *r*_DC_. That was because, unlike the tectorial membrane/hair bundle motion, both structures (outer hair cell and Deiters cell) rotated about a similar point (the inner pillar cell root). According to model simulations, *r*_DC_ values to obtain measured θ_DC_ ranged anywhere between 1 and 10.

### Other OoC properties affecting the motion of outer hair cell/Deiters cell joint

The phalangeal process of Deiters cell forms a Y-shaped structure together with the outer hair cell and the body of Deiters cell (Fig. 2*B*). Considering such an arrangement, the phalangeal process can affect force transmission from the outer hair cell (Yoon et al., 2011; Bormuth et al., 2014). Based on experiments with isolated Deiters cells, the Young's modulus of the phalangeal process has been estimated to be 45 ± 35 MPa (Laffon and Angelini, 1996). Our default value of 40 MPa corresponds to the axial and bending stiffness of 200 and 0.01 times the outer hair cell stiffness, which were obtained from EA/L and $3EI_{z}/L^{3}$, respectively. E, $I_{z}$, and L are the Young's modulus, second area moment of inertia, and length, respectively. The phalangeal process stiffness (*k*_PhP_) had a modest effect on the motion of outer hair cell/Deiters cell joint; as *k*_PhP_ varied a hundred times, Δ_DC_/Δ_OHC_ changed four times.

The reticular lamina is a tile-like composite consisting of the apical parts of outer pillar cells, hair cells, and phalangeal processes. These tiles, packed with microtubules or actin fibers, are tightly bound by junction proteins to form the reticular lamina. Thus the reticular lamina is likely stiff. According to FE model analysis, Δ_DC_/Δ_OHC_ was minimally affected by the change of reticular lamina stiffness (Fig. 9*C*). Meanwhile, the directions of outer hair cell/Deiters cell joint motion (θ_DC_) were affected by *k*_RL_, when *k*_RL_/*k*_OHC_ ≈ 1 (Fig. 9*D*). Here, the reticular lamina stiffness was represented by the bending stiffness of $3EI_{z}/L^{3}$. The reticular lamina with its large axial stiffness value (>10^3^ *k*_OHC_) can be considered rigid. In our model, the reticular lamina was clamped to the pillar cell top. An alternative condition was examined—a rigid reticular lamina freely rotates about the pillar cell top. Under such a condition, we could not find a parameter set that could explain the observed OoC motion. Our measured results were consistent with a stiff reticular lamina (*k*_RL_/*k*_OHC_ ≫ 1). Therefore, the reticular lamina (at least until the first-row outer hair cell) can be considered a stiff cantilever beam sticking out of the top plate of the tunnel of Corti.

The viscous damping of OoC structures had minimal effect on the OoC vibrating pattern. However, the damping property of Deiters cell and outer hair cell could affect θ_DC_ when the damping property was much greater than the default value used in our model (Fig. 9*F*, indicated with symbol ▾). For both the outer hair cell and the Deiters cell, the dissipating properties used in our FE model was 5 nN·s/m per 10 µm cell length, which were 27 and 34 nN · s/m at *x* = 8.5 mm, for the respective cells. These damping values can be compared with relevant properties in the literature. In cochlear model studies, the damping coefficient is on the order of 1 kN·s/m per unit area of the basilar membrane (Prodanovic et al., 2019, their Table 3). Considering the basilar membrane dimensions (300 µm wide and 10 µm section), this value of 1 kN·s/m^3^ corresponds to 300 nN·s/m. Often, dissipation in the subtectorial space represents OoC complex damping. The equivalent damping coefficient of the subtectorial space is on the order of 100 nN·s/m.

## Discussion

Our approach features anatomic details both in measurements and model simulations of the OoC vibrations. Individual hair cells in the OoC were imaged from which their motion was measured (Fig. 1*E*,*F*). The FE model took advantage of acquired anatomic information (Fig. 2*E–H*). Computer simulations reproduced measured responses to both hydrostatic and hydrodynamic stimulations (Figs. 3, 6). We focused on passive mechanical responses across the OoC radial section rather than the more extensively investigated subject of active traveling waves along the length of the cochlea. The combined approach enabled us to quantify mechanical attributes of the OoC complex that have been challenging to acquire, such as the radial stiffness of the tectorial membrane and the axial stiffness of the Deiters cell. In the following, we discuss how our findings from passive micromechanics provide insights on force transmission in the OoC.

### Tectorial membrane attachment (radial) stiffness

The role of the tectorial membrane has been an inspiring topic. A decades-old theory is that the tectorial membrane serves as a second resonator (the first one being the basilar membrane; (Zwislocki and Kletsky, 1979; Gummer et al., 1996). Another theory considers the tectorial membrane as the medium to carry the second layer of traveling waves (Ghaffari et al., 2007; Sellon et al., 2017). The observation of sharper frequency tuning of the mouse cochlea with genetic defects in the tectorial membrane partly supports these theories (Russell et al., 2007). Previous studies showed that the model responses change qualitatively depending on tectorial membrane mechanical properties (Meaud and Grosh, 2010; Nam and Fettiplace, 2010; Meaud and Grosh, 2014; Liu et al., 2015, 2017; Nankali et al., 2020).

The mechanical properties of the tectorial membrane have been measured by different research groups using various methods. Notably, most of those measurements were performed *ex situ* (after detaching the tectorial membrane from the OoC), although the attachment (radial) stiffness is critical to examine existing theories. Exceptionally, two studies measured the tectorial membrane stiffness *in situ*. Zwislocki and Cefaratti (1989) measured radial stiffness of the tectorial membrane. However, with a displacement amplitude over tens of micrometers, the tectorial membrane was likely detached from the OoC, and it could have bent or buckled. Richter et al. (2007) also measured the tectorial membrane stiffness *in situ* (Teudt and Richter, 2014). They measured flexural rigidity on the tectorial membrane side of the OoC complex, and the tectorial membrane also was deformed tens of micrometers. Arguably, our study is the first to report the radial stiffness of the tectorial membrane that remains attached to the spiral limbus and hair bundles. Our results indicate that the attachment of the tectorial membrane is approximately twice as stiff as the hair bundle. For the measured locations (7.5 and 8.5 mm from the basal end of the gerbil cochlea), the estimated stiffness value is 9–12 mN/m per 10 µm section. This corresponds to the Young's modulus of 2.5–3.0 kPa in the radial direction.

### Deiters cells are the equalizer for OoC mechanics

For our FE model to be comparable to the measured motion of OoC structures, the reticular lamina and the pillar cells had to be much stiffer than the outer hair cell. Meanwhile, the Deiters cell that is in series with the outer hair cell had to have a stiffness comparable to that of the outer hair cell. We argue that these conditions (stiff and not-stiff surrounding structures of the outer hair cells) are required to perform two mechanical functions of the OoC—active and passive power transmission.

**Figure 10.**
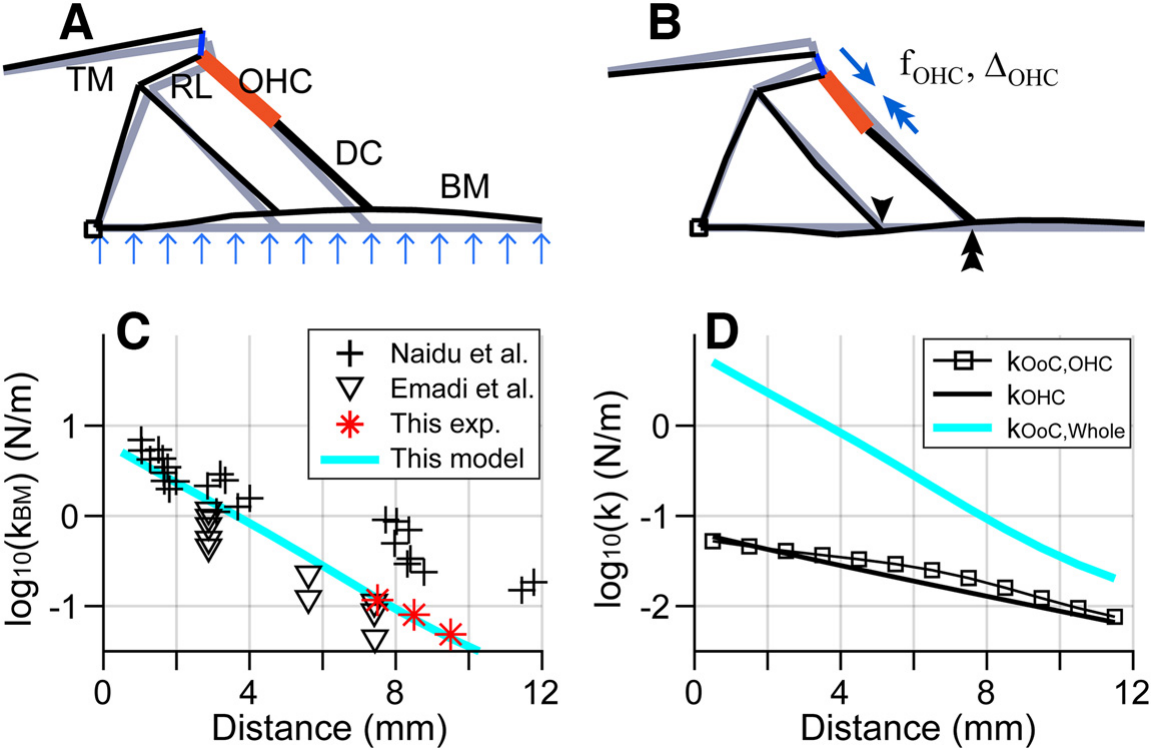
Stiff yet deformable OoC for passive and active force transmission. ***A***, Passive force transmission, simulated deformation pattern because of fluid pressure. ***B***, Active force transmission, simulated deformation pattern because of the contractile force of outer hair cells; f_OHC_ is an equal and opposite force couple applied at the ends of outer hair cell along the axis of the cell (paired blue arrow). Δ_OHC_ is the resulting deformation of the cell. The stiffness of OoC supporting structures felt by the outer hair cell is *k*_OoC,OHC_ = *f*_OHC_/Δ_OHC_ − *k*_OHC_, where *k*_OHC_ is the axial stiffness of the outer hair cell. The basilar membrane was displaced minimally because of counteracting deformations (single and double arrowheads). ***C***, Contact stiffness at the basilar membrane. The stiffness values of two previous studies are shown together with the measured and simulated values in this study. ***D***, Stiffness gradient obtained from FE model simulations. Stiffness of the whole OoC complex (*k*_OoC,Whole_), *k*_OHC_ and *k*_OoC,OHC_. The values are per 10 µm sections.

Appropriate compliance of the Deiters cell is required for active and passive power transmission. Our simulated and measured vibration patterns (rotating motion about the inner pillar cell root, Figs. 5, 10*A*) are consistent with the widely accepted notion of stiff tunnel of Corti. Meanwhile, the Deiters cell was neither too stiff nor too compliant compared with the outer hair cell; the Deiters cell was two to four times stiffer than the outer hair cell body (Fig. 8).

Our modeled and measured stiffness of the OoC complex are summarized in Figure 10, *C* and *D*. Measured volume compliance was translated into contact stiffness (Fig. 10*C*, asterisks) by a two-step process: First, the elastic property of the FE model (Young's modulus of the basilar membrane) was adjusted to match the measured volume compliance. Then, contact forcing was simulated to obtain the relevant contact stiffness. In Figure 10*D* the whole OoC complex stiffness (thick green curve, *k*_OoC,Whole_) along the cochlear length was compared with outer hair cell stiffness (black curve, *k*_OHC_) and the stiffness felt by an outer hair cell (square markers, *k*_OoC,OHC_); $k_{OoC,OHC}$ was obtained from the relation between outer hair cell force and OoC deformation $k_{OoC,OHC}=f_{OHC}/\Delta_{OHC}-k_{OHC}$ (Liu et al., 2017). Although *k*_OoC,Whole_ varies three orders of magnitude over the cochlear length, $k_{OHC}$ changes about one order of magnitude. Despite the discrepancy in the stiffness gradient between the overall stiffness and outer hair cell, the stiffness felt by the outer hair cell (*k*_OoC,OHC_) remained comparable to that of the outer hair cell. When the Deiters cell stiffness was two to three times the outer hair cell stiffness, the stiffness felt by an active outer hair cell remained close to its own stiffness over the entire cochlear length (*k*_OoC,OHC_/*k*_OHC_ = 0.5–1.5).

### Basilar membrane serves as a mechanical reference for outer hair cell motility

Our results provide a quantitative basis for classical OoC kinematics as well as those previous studies that recognized the mechanical role of Deiters cells in active cochlear function (Nam, 2014; Wang et al., 2016; Sasmal and Grosh, 2019; van der Heijden and Vavakou, 2022). When information on a deformable OoC was lacking, a reasonable simplification was that two elastic media (tectorial and basilar membranes) are interconnected by a link (stereocilia) sitting on a rigid structure (the tunnel of Corti). Relative motion between two rotating bodies results in the deflection of the stereociliary bundle. Our observations and model simulations support this classical understanding (Figs. 5*A*, 6*A*, 10*A*). Meanwhile, recent observations of sensitive cochleas show that OoC substructures do not vibrate in phase and that outer hair cells and their vicinity vibrate more than the basilar membrane (Lee et al., 2016; Cooper et al., 2018; He et al., 2018; Strimbu et al., 2020). Our previous study with the same preparation method as the present study (Jabeen et al., 2020) also showed vibration patterns similar to sensitive cochleas when the OoC was vibrated by outer hair cell motility.

Figure 10*B* is the simulated deformation pattern when the OoC is subjected to outer hair cell contraction. The contraction was represented by a pair of equal and opposite forces at the ends of the outer hair cell. This snapshot of force transmission demonstrates how the supporting structures of outer hair cell (tunnel of Corti and Deiters cell) shapes the OoC deformation pattern because of outer hair cell motility. There are two routes of force transmission. The pillar cell triangle is pushed down (single arrowhead), whereas the Deiters cell is pulled up (double arrowhead) by outer hair cell contraction. Those two force transmissions deflect the basilar membrane in the opposite directions. As a result, overall transverse displacement of the basilar membrane remains minimal, whereas the reticular lamina is subjected to substantial downward displacement. Our results provide a physical explanation for recent observations of how outer hair cell motility generates greater vibrations at the top side of the OoC than its bottom side (Warren et al., 2016; Cooper et al., 2018; He et al., 2018). Our simulated observation predicts a higher-order mode in basilar membrane vibrations when outer hair cell motility is prominent.
